# Supplementary material for: Rel Family Transcription Factor NFAT5 Upregulates COX2 via HIF-1α Activity in Ishikawa and HEC1a Cells
Source: Int J Mol Sci. 2024 Mar 25;25(7):3666. doi: 10.3390/ijms25073666 (PMC11012216; doi:10.3390/ijms25073666)
Supplement: Supplementary file 1 [file ijms-25-03666-s001.zip › Supplementary Information_25032024.pdf]

Rel family transcription factor NFAT5 upregulates COX2 *via* HIF-1α activity in Ishikawa and HEC1a cells

Toshiyuki Okumura[1,2], Janet Raja Xavier[1], Jana Pasternak[1], Zhiqi Yang[1], Cao Hang[1], Bakhtiyor Nosirov[4], Yogesh Singh[1,3], Jakob Admard[3], Sara Y. Brucker[1], Stefan Kommoss[1], Satoru Takeda[2], Annette Staebler[5], Florian Lang[6] and Madhuri S. Salker[1]*

Supplementary Information

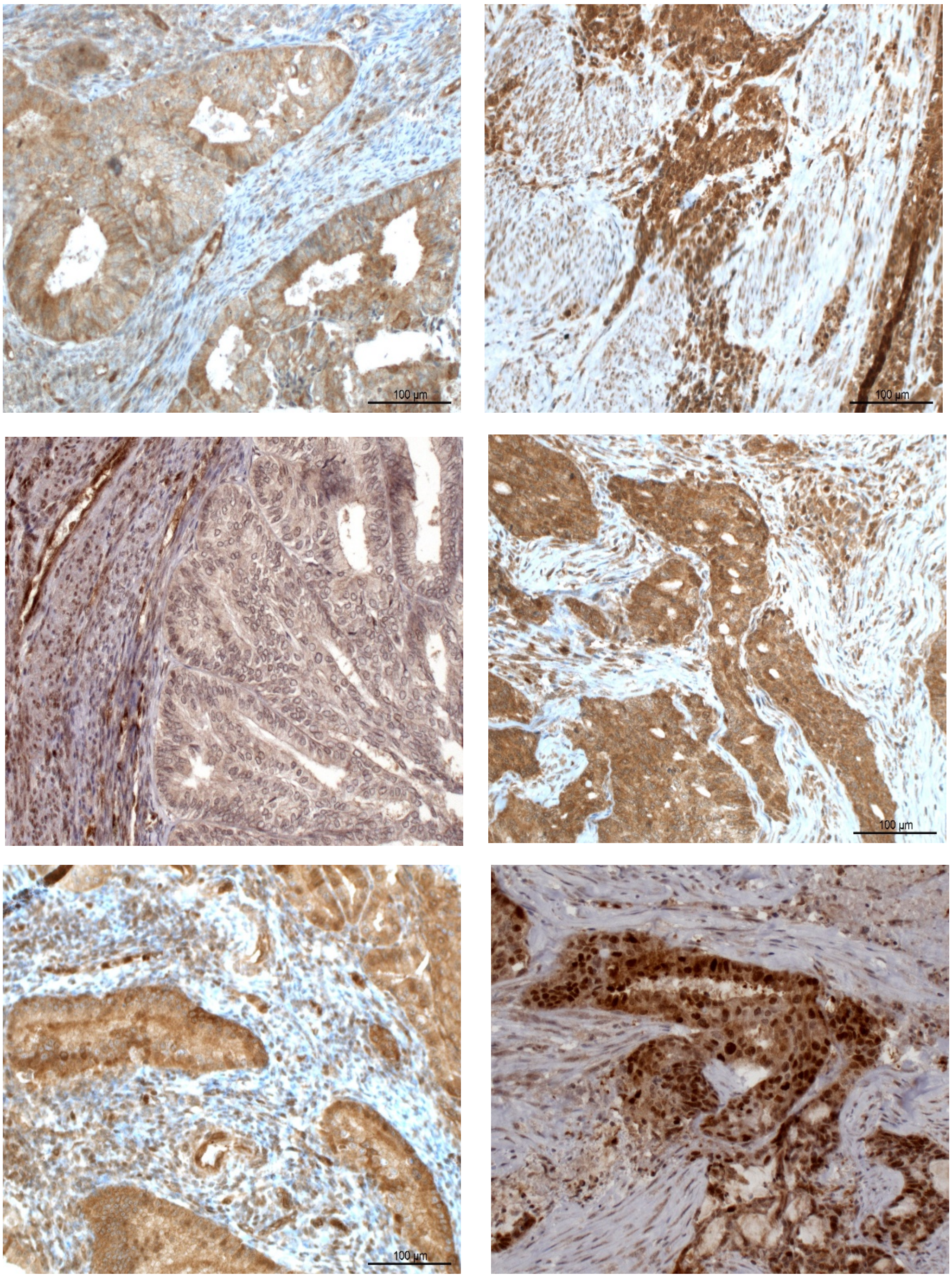

Figure S1. Representative immunohistochemistry images of NFAT5 staining in cases of grade 1 and grade 3 adenocarcinomas.

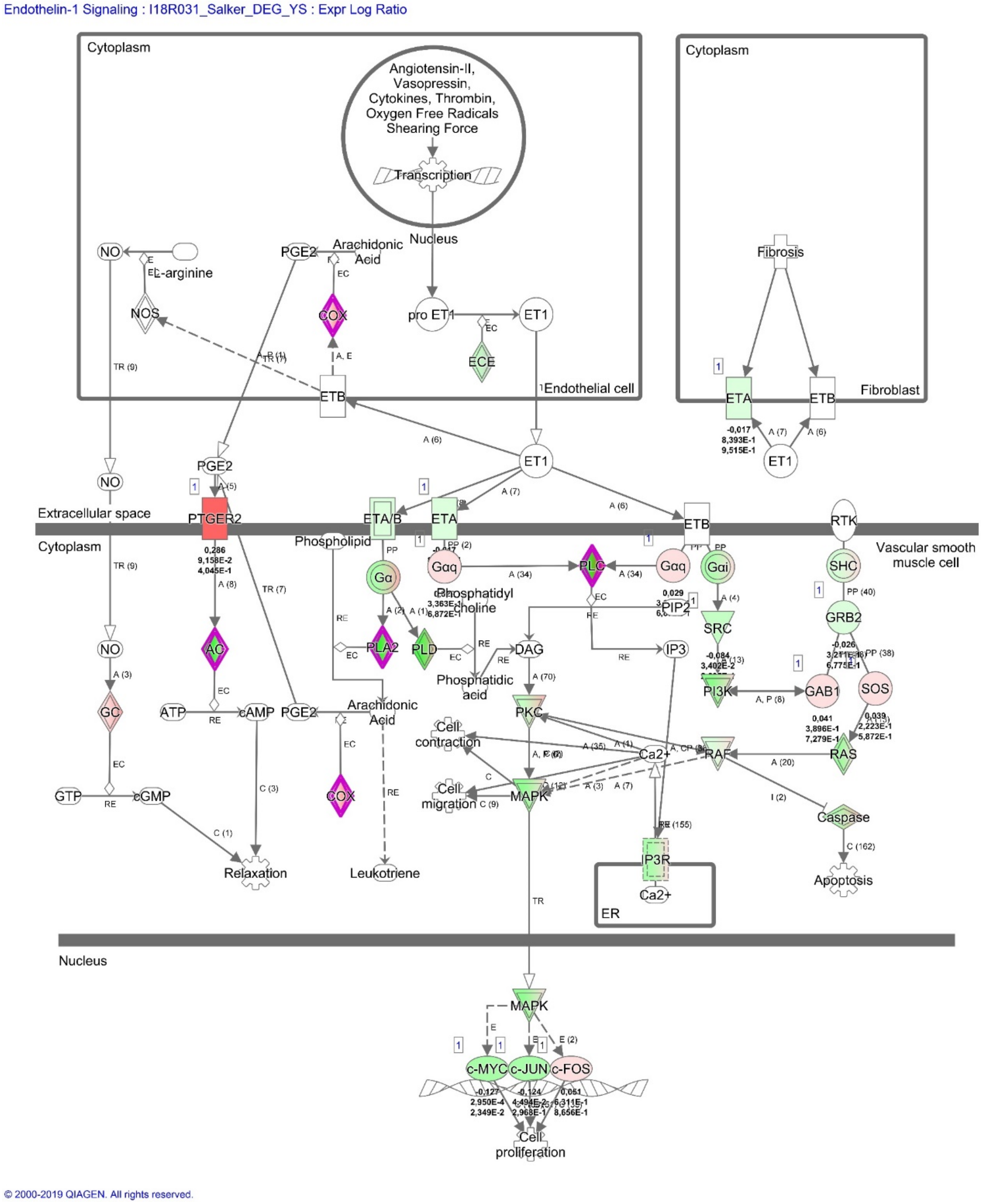

Figure S2. QIAGEN Ingenuity Pathway Analysis with RNA sequencing data (Con/NFAT5 overexpressed) from Ishikawa cells revealed the associated cellular signaling pathway activated. NFAT5 overexpression points to the activation of PTGS2 (COX2) signaling.

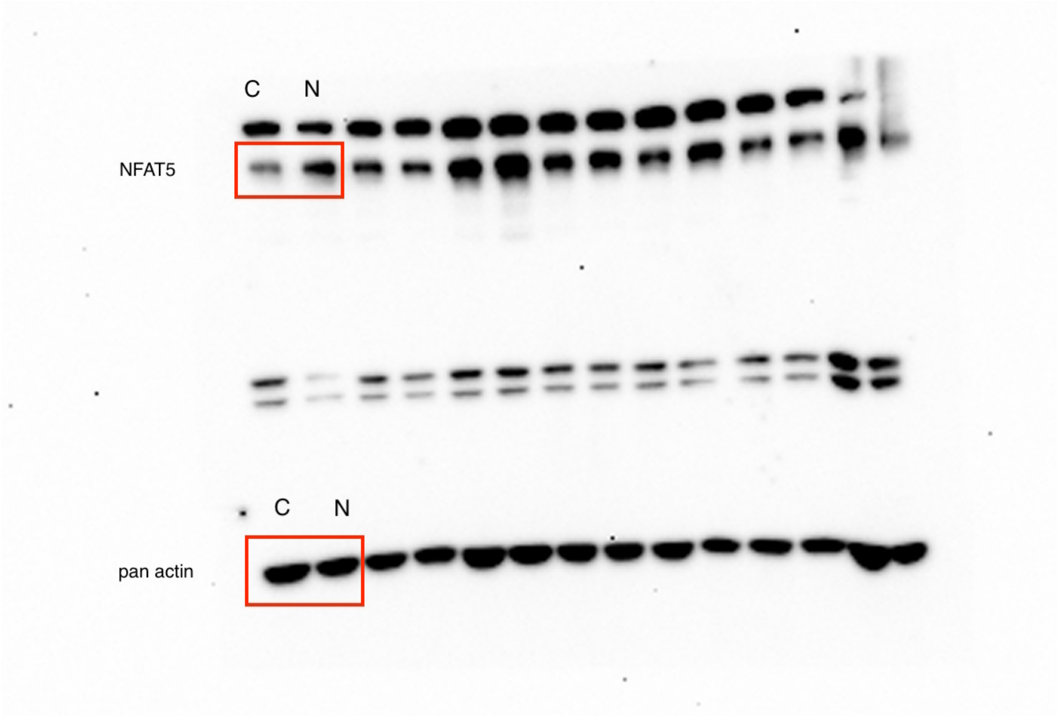

Figure S3. Original western blot membranes of blots represented in figure 3.

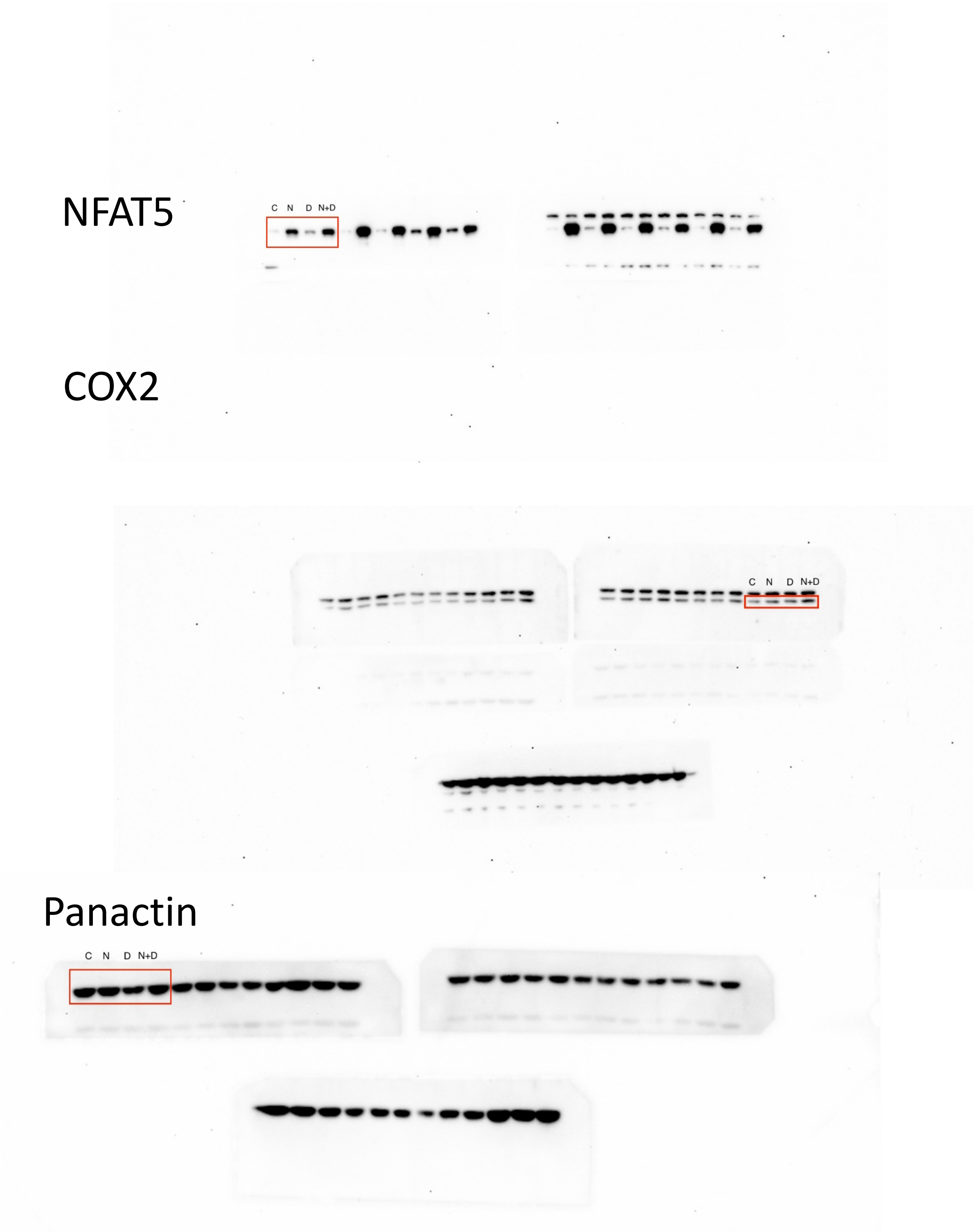

Figure S4. Original western blot membranes of blots represented in figure 5.

(a)

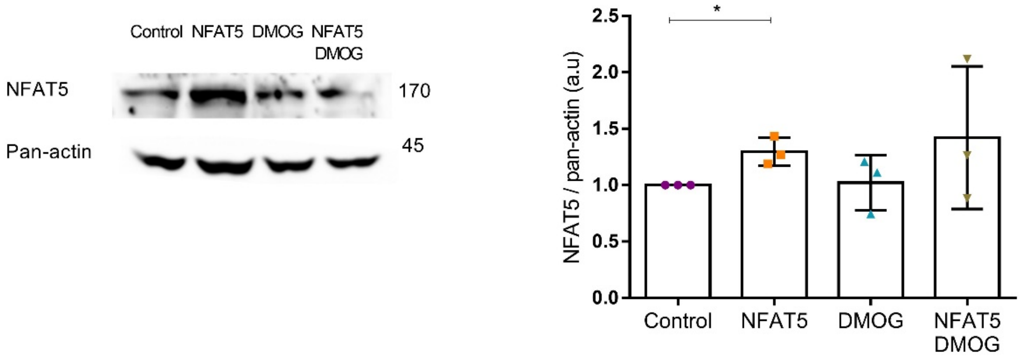

(b)

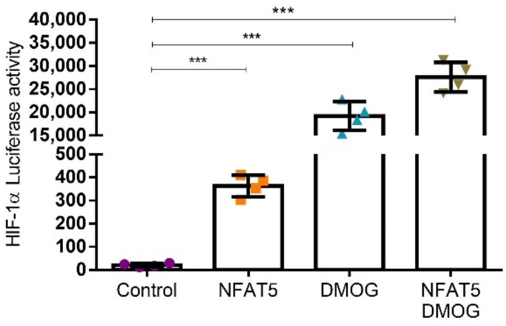

(c)

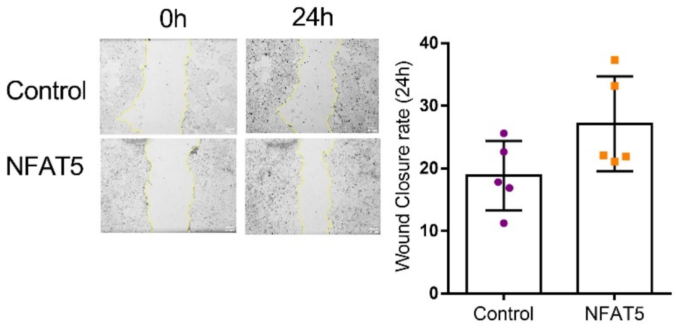

Figure S5. Effect of NFAT5 overexpression in HEC1a cells. HEC1a cells were treated with 0.5 mM DMOG for 24 hours, after 24 hours transfection with NFAT5 overexpression plasmid. (a). NFAT5 protein abundance were investigated by western blot analysis (n=3, *, P, <0.05). (b) HIF-1α promoter activation upon NFAT5 transfection in verified with luciferase reporter assay (n=4, ***, P<0.001). (c) Effect of NFAT5 overexpression on HEC1a cell migration was verified with wound healing scratch assay (n=5). Representative bright field images and wound closure rate 24 hours post scratch.
